# Dataset of proinflammatory cytokine and cytokine receptor gene expression in rainbow trout (*Oncorhynchus mykiss*) measured using a novel GeXP multiplex, RT-PCR assay

**DOI:** 10.1016/j.dib.2017.02.014

**Published:** 2017-02-12

**Authors:** Ivan Kutyrev, Beth Cleveland, Timothy Leeds, Gregory D. Wiens

**Affiliations:** a National Center for Cool and Cold Water Aquaculture, 11861 Leetown Rd, Kearneysville, WV 25430, USA; b Institute of General and Experimental Biology, Siberian Brunch of Russian Academy of Sciences, Sakhyanovoi St., 6, 670047 Ulan-Ude, Russia

**Keywords:** *Flavobacterium psychrophilum*, Cytokine, Disease resistance, Bacterial cold water disease, Rainbow trout

## Abstract

A GeXP multiplex, RT-PCR assay was developed and optimized that simultaneously measures expression of a suite of immune-relevant genes in rainbow trout (*Oncorhynchus mykiss*), concentrating on tumor necrosis factor and interleukin-1 ligand/receptor systems and acute phase response genes. The dataset includes expression values for *drpt*, *il11a*, *il1b1*, *il1b2*, *il1b3*, *il1r-like-1*(e3-5), *il1r-like-1*(e9-11), *il1r1-like-a*, *il1r1-like-b*, *il1r2*, *saa*, *tnfa1*, *tnfa2*, *tnfa3*, *tnfrsf1a*, *tnfrsf1a-like-a*, *tnfrsf1a-like-b*, *tnfrsf5*, and *tnfrsf9*. Gene expression was measured at four time-points post-challenge in both a resistant line (ARS-Fp-R) and a susceptible line (ARS-Fp-S) of rainbow trout. In addition, fish body weight, spleen index and the *Flavobacterium psychrophilum* load are reported. These data are an extension of information presented and discussed in **“**Proinflammatory cytokine and cytokine receptor gene expression kinetics following challenge with *Flavobacterium psychrophilum* in resistant and susceptible lines of rainbow trout (*Oncorhynchus mykiss*)” (Kutyrev et al., 2016) [1].

**Specification Table**

**Table:** 

Subject area	Biology
More specific subject area	Immunogenetics
Type of data	Tables, figures
How data was acquired	GeXP multiplex, RT-PCR assay (Genome Lab GeXP genetic analysis system; Beckman Coulter Inc.; Pasadena, CA, USA). *qPCR* (ABI 7900HT real-time thermal cycler; Applied Biosystems).
Data format	Raw
Experimental factors	Challenge: *Flavobacterium psychrophilum* strain CSF259-93 or PBS
	Sampling timepoints: 6 h, 24 h, 48 h and 144 h post-injection
	Genetic line: ARS-Fp-R (resistant) and ARS-Fp-S (susceptible)
	Tanks: 1–8
	RNA extraction batch: 1–8
	GeXP run: 1–3
Experimental features	Tanks were randomly assigned to treatment. Fish were randomly assigned to tanks by genetic line. For sampling, PBS injected fish were sampled first, prior to sampling infected animals to prevent cross-contamination.
Data source location	National Center for Cool and Cold Water Aquaculture, 11861 Leetown Rd, Kearneysville, WV 25430, USA
Data accessibility	Data are within this article

**Value of the data**

• Data are provided describing the optimization and validation of a new GeXP assay for measuring rainbow trout proinflammatory cytokines.
• The data include PCR product relative migration and identification that are of use to researchers when performing this assay.
• The gene expression values from the rainbow trout resistant line, ARS-Fp-R, and the susceptible line, ARS-Fp-S, can be used in future studies for meta-analyses comparing the response to other pathogens or environmental conditions.

## 1 Data

**Fig. 1:**
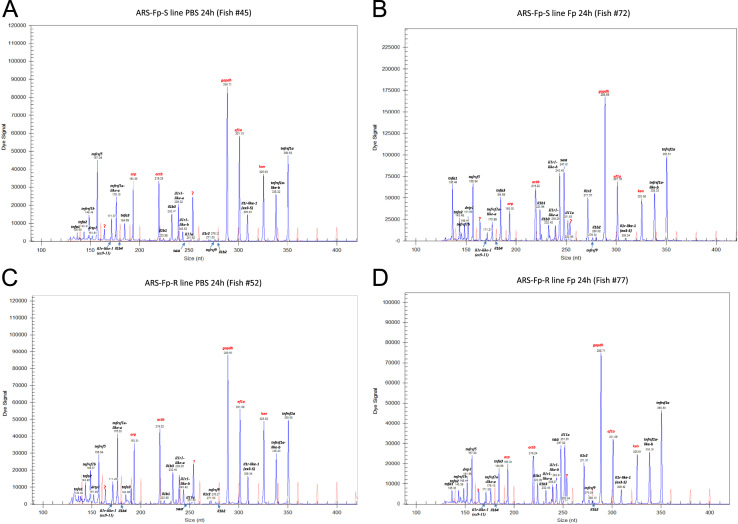
Dye signal, size resolution and identification of PCR products from the GeXP assay. Representative spleen sample amplification samples: (A) ARS-Fp-S line fish #45 injected with PBS and tissue harvested at 24 h, (B) ARS-Fp-S line fish #72 injected with *Fp* and tissue harvested at 24 h, (C) ARS-Fp-R line fish #52 injected with PBS and tissue harvested at 24 h, and (D) ARS-Fp-R line fish #77 injected with *Fp* and tissue harvested at 24 h. Chromatogram lines shown in red are size standards. Genes labeled in red font are reference genes as well as the kanamycin^r^ positive control.

**Fig. 2:**
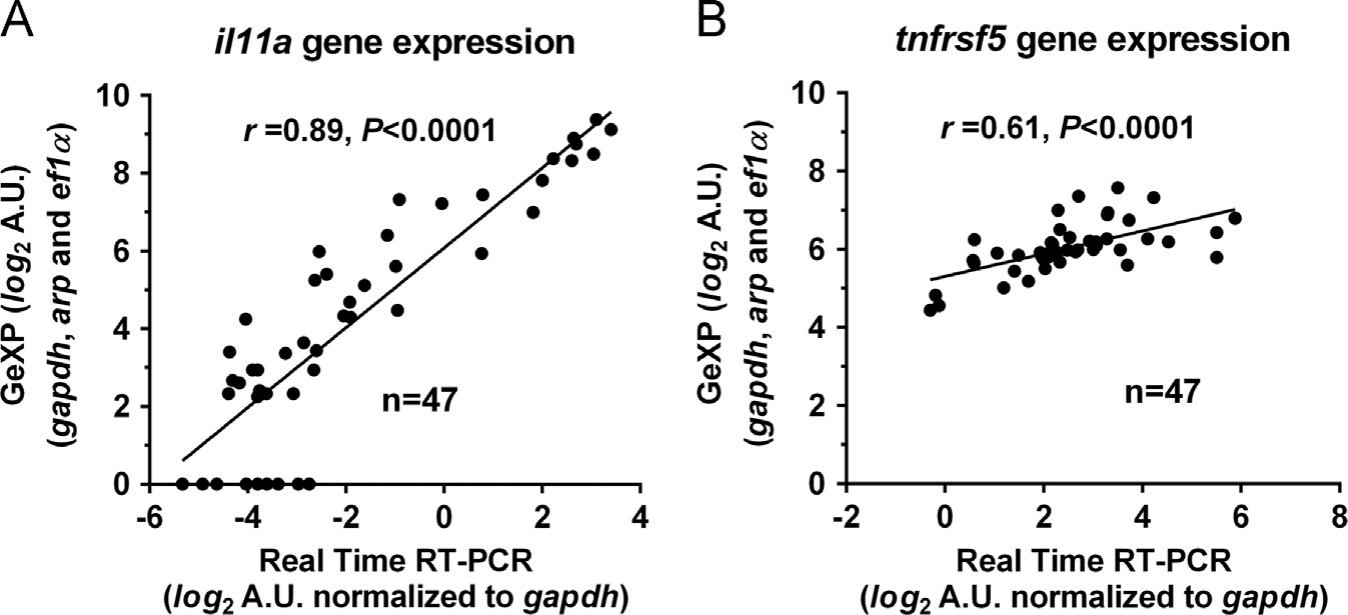
Comparison of gene expression measured by GeXP and real time RT-PCR for *il11a* (A) and *tnfrsf5* (B). A total of 47 samples were compared that represented 2–4 samples from each line, treatment and timepoint.

The dataset includes assay optimization and meta-data associated with measuring gene expression kinetics of bacterial cold water disease resistant and susceptible genetic lines of rainbow trout (*Oncorhynchus mykiss*) following challenge with *Flavobacterium psychrophilum* [1]. The GeXP assay primer dilutions were optimized and the predicted PCR products matched their elution peak in single reactions (Table 1). Kanamycin gene positive control RNA was included as an internal control for the RT and PCR reactions. Spleen samples were collected at 6 h, 24 h, 48 h and 144 h post-challenge, RNA extracted and reverse transcribed, and gene expression measured by GeXP [1]. Representative amplification profiles from 24 h samples are shown (Fig. 1A–D). Two unidentified bands were present at 253 bp and 163 bp. The complete gene expression dataset includes the meta-data of tank number, sample order, RNA extraction batch, GeXP run and normalized gene expression value (Supplemental Data Table 1). Correlation of gene expression values measured either GeXP or by syber green RT-PCR or for genes *il11a* (Fig. 2A) and *tnfrsf5* (Fig. 2B). Expression of *tnfrsf5* was not regulated by infection [1] and thus has lower variation between samples and a lower correlation between assays [2].

## 2 Experimental design, materials and methods

### 2.1 Gene expression analysis

The Genome Lab GeXP genetic analysis system (Beckman Coulter Inc.; Pasadena, CA, USA) was used to quantify transcript expression. Primers were designed as described [1]. Amplicons were between 100 and 400 base pairs and each PCR product was separated in size by a minimum of three base pairs (Table 1). Twenty-one primer-pairs amplified genes associated with the immune response and four primer pairs amplified reference genes (Table 1). The multiplex reverse transcription (RT) primer concentrations were optimized by dilution for spleen tissue (Table 1). The size of each amplicon closely matched the expected length (Table 1).

### 2.2 Real time RT-PCR

One microgram of DNase treated RNA was used in a 40 μL reverse transcription reaction random primers (Invitrogen, Carlsbad, CA) and MMLV (Promega, Madison, WI) per manufacturer׳s protocol. Four μL of cDNA was used in a 15 µL PCR reaction with 7.5 µL 2X SYBR Green Master Mix (ABI Biosystems, Foster City, CA), 833 nM forward primer and 833 nM reverse primer. Real-time PCR reactions were completed in duplicate for *tnfrsf5* and *il11a*, with *gapdh* as a reference gene. Primers sequences were identical to those used in the GeXP multiplex assay. The real time PCR was performed with an ABI7900 Sequence Detection System (Applied Biosystems) using a 2-step PCR procedure with the following protocol. Temperature was held at 50 °C for 2 min followed by 95 °C for 10 min. Forty PCR cycles of 95 °C (15 s) and 60 °C (1 min) were completed, followed by melt curve analysis to confirm a single PCR product per reaction. PCR efficiencies were initially determined using a 6–10-step two-fold serial cDNA dilution. PCR efficiencies were 2.05, 2.08, and 2.19 for *tnfrsf5*, *il11a*, and *gapdh*, respectively.

## Figures and Tables

**Table 1:** Gene product predicted size and elution peak size determined using optimized primer concentrations. The trout reference genes are *arp*, *actb, gapdh* and *ef1α*. Kan^r^ RNA is an independent template used as a positive control for RT and PCR reactions.

Gene Product	Predicted Size (bp)	Elution Peak (bp)	Primer dilution (500 nM, 1:1)						
			2:1	1:1	1:2	1:4	1:8	1:16	1:32
*saa*	247	247.8						X	
*drtp1*	151	151.9	X						
*il1b1*	223	223.9					X		
*il1b2*	279	280.1		X					
*il1b3*	232	232.4		X					
*il1b4*	179	N.D.		X					
*il11a*	251	251.6		X					
*il1r-like-1(ex 9–11)*	168	171.4				X			
*il1r-like-1(ex 3–5)*	307	309.4	X						
*il1r1-like-a*	238	239.3		X					
*il1r1-like-b*	241	243.4	X						
*il1r2*	270	271.5		X					
*tnfa1*	136	136.4						X	
*tnfa2*	143	143.4		X					
*tnfa3*	184	184.7		X					
*tnfrsf1a*	349	350.4		X					
*tnfrsf1a-like-a*	175	176.1				X			
*tnfrsf1a-like-b*	337	338.2		X					
*tnfrsf1b*	147	148.4		X					
*tnfrsf5*	155	156.9				X			
*tnfrsf9*	275	276.3	X						
*arp*	192	193.3							X
*actb*	218	219.2							X
*gapdh*	287	288.7		X					
*ef1α*	301	301.1						X	
Kan	325	325.6				X			
