# Supplementary material for: Dataset of proinflammatory cytokine and cytokine receptor gene expression in rainbow trout (Oncorhynchus mykiss) measured using a novel GeXP multiplex, RT-PCR assay
Source: Data Brief. 2017 Feb 12;11:192–6. doi: 10.1016/j.dib.2017.02.014 (PMC5315433; doi:10.1016/j.dib.2017.02.014)
Supplement: Supplementary file 1 — Supplementary material [file mmc1.docx]

January 19, 2017

Dr. Marina Ostankovitch, PhD

Senior Scientific Editor

Data in Brief

Dear Dr. Ostankovitch:

Conflicts of interest: none

Sincerely,

Gregory D. Wiens, PhD.

Research Immunologist and Lead Scientist
